# Multimorbidity and health-related quality of life (HRQoL) in a nationally representative population sample: implications of count versus cluster method for defining multimorbidity on HRQoL

**DOI:** 10.1186/s12955-016-0580-x

**Published:** 2017-01-09

**Authors:** Lili Wang, Andrew J Palmer, Fiona Cocker, Kristy Sanderson

**Affiliations:** 1 Menzies Institute for Medical Research, University of Tasmania, 17 Liverpool Street, Hobart, TAS 7000 Australia; 2 Monash Centre for Occupation and Environmental Health (MonCOEH), Department of Epidemiology and Preventive Medicine, Monash University, Melbourne, Australia; 3 School of Health Sciences, Faculty of Medicine and Health Sciences, University of East Anglia, Norwich, UK

**Keywords:** Multimorbidity, Definition, AQoL-4D, Hierarchical cluster, Health-related Quality of Life (HRQoL)

## Abstract

**Background:**

No universally accepted definition of multimorbidity (MM) exists, and implications of different definitions have not been explored. This study examined the performance of the count and cluster definitions of multimorbidity on the sociodemographic profile and health-related quality of life (HRQoL) in a general population.

**Methods:**

Data were derived from the nationally representative 2007 Australian National Survey of Mental Health and Wellbeing (*n* = 8841). The HRQoL scores were measured using the Assessment of Quality of Life (AQoL-4D) instrument. The simple count (2+ & 3+ conditions) and hierarchical cluster methods were used to define/identify clusters of multimorbidity. Linear regression was used to assess the associations between HRQoL and multimorbidity as defined by the different methods.

**Results:**

The assessment of multimorbidity, which was defined using the count method, resulting in the prevalence of 26% (MM2+) and 10.1% (MM3+). Statistically significant clusters identified through hierarchical cluster analysis included heart or circulatory conditions (CVD)/arthritis (cluster-1, 9%) and major depressive disorder (MDD)/anxiety (cluster-2, 4%). A sensitivity analysis suggested that the stability of the clusters resulted from hierarchical clustering. The sociodemographic profiles were similar between MM2+, MM3+ and cluster-1, but were different from cluster-2. HRQoL was negatively associated with MM2+ (β: −0.18, SE: −0.01, *p* < 0.001), MM3+ (β: −0.23, SE: −0.02, *p* < 0.001), cluster-1 (β: −0.10, SE: 0.01, *p* < 0.001) and cluster-2 (β: −0.36, SE: 0.01, *p* < 0.001).

**Conclusions:**

Our findings confirm the existence of an inverse relationship between multimorbidity and HRQoL in the Australian population and indicate that the hierarchical clustering approach is validated when the outcome of interest is HRQoL from this head-to-head comparison. Moreover, a simple count fails to identify if there are specific conditions of interest that are driving poorer HRQoL. Researchers should exercise caution when selecting a definition of multimorbidity because it may significantly influence the study outcomes.

## Background

The presence of multiple chronic conditions, also known as multimorbidity, is in the health care spotlight due to its increasing prevalence, complex management and large economic disease burden [1, 2]. Approximately 25% adults have at least two chronic conditions, and more than half the elderly have three or more conditions simultaneously [3]. Although the prevalence of multimorbidity is higher among adults aged 65 years and over, more than half of individuals with multimorbidity are younger than 65 years [4, 5], which makes multimorbidity an issue across the lifespan.

Health-related quality of life (HRQoL) is a holistic concept that aims to capture a range of health status indices. To date, the impact of multimorbidity on HRQoL has been investigated based on two general categories of multimorbidity: i) the number of chronic conditions (count definition) and ii) the cluster of chronic conditions (cluster definition) [6, 7]. Although HRQoL scores decrease with an increasing number of co-occurring chronic conditions [8], the full impact of multimorbidity on HRQoL is unlikely to be captured by the simple count method [9]. Meanwhile, some specific clusters of multimorbidity, such as the combination of mental and physical conditions [10], have been shown to have a notable effect on HRQoL. However, the impact of the different definitions of multimorbidity on HRQoL in a primary care setting is still unclear [8].

Comparing how the aforementioned categorizations of multimorbidity effect the sociodemographic profile and health status (HRQoL) will provide a conclusive definition of multimorbidity, and consequently, improve health care planning in the context of multimorbidity to match healthcare services with patients’ needs. Therefore, using a large, nationally representative dataset, this study examined the performance of the count and cluster definitions of multimorbidity in determining the sociodemographic profile and HRQoL in a general population.

## Methods

### Study design and participants

Our study was a cross-sectional analysis of a nationally representative dataset, the 2007 National Survey of Mental Health and Wellbeing (NSMHWB), which consisted of a series of face-to-face interviews conducted by the Australian Bureau of Statistics (ABS) from August to December 2007. Respondents were randomly selected from a stratified, multistage area probability sample of respondents’ homes. More methodological information could be found elsewhere [11]. There were 14,805 eligible dwellings out of an initial sample of 17,352 dwellings due to all household members being out of scope or vacant dwellings. Of these, the final data set consisted of 8,841 respondents (60% response rate) aged 16 to 85 years of age and living in private dwellings [12]. No missing data strategy was used due to the low rate of missing data (2.6%): 21 due to no HRQoL score, 34 due to log-transformed HRQoL score, 180 due to BMI and 6 due to exercise level.

### Multimorbidity

Multimorbidity was identified from a pre-specified list including the following self-reported conditions that significantly contribute to the global burden of illness and injury [12–14]: asthma, cancer, stroke, heart or circulatory conditions (CVD), gout, rheumatism or arthritis, diabetes or high sugar levels, major depressive disorder (MDD) and anxiety disorder (including agoraphobia, with or without panic disorder, generalized anxiety disorder (GAD) and social phobia). Each chronic condition was coded as present or absent [12]. The diagnosis of mental disorders was established using the World Mental Health Survey Initiative version of the World Health Organization's Composite International Diagnostic Interview, version 3.0 (WMH-CIDI 3.0) [14], which is a comprehensive and fully structured diagnostic interview. The timeframe was a diagnosis in the 12 months prior to the interview. Diagnosis of physical chronic conditions was determined from a pre-specified list by whether the respondent had ever been told by a doctor or nurse that they had these conditions, and stroke was assessed using self-reported stroke symptoms [11].

In the count method, multimorbidity was defined as “two or more” chronic conditions occurring at the same time. To test the validation in cut-off of count based method, the definition of multimorbidity “having 3+ chronic conditions at the same time in one individual” (known as complex multimorbidity) [15] was used as well. In the cluster-based method, hierarchical clustering was used to identify the common clusters of multimorbidity as chronic health conditions can co-occur via some sharing underlying genetic, environmental, or behavioural risk factors [16–18]. Assuming N variables, the hierarchical approach initially treated each variable as a cluster before merging the two closest variables into a new cluster. This step was repeated until all variables were merged into one cluster of size N. Jaccard’s coefficient was used to calculate the distance of the binary variables (absence or presence of conditions) [16, 19]. The results may vary depending on the different distance calculation methods. Both Ward’s and the average linkage methods have been widely used, with the former considered more appropriate for clusters with equal numbers of observations [19] and the latter recommended to avoid large or tight compact clusters that result from the single linkage and the complete linkage methods [19]. Therefore, we used the average linkage method in this study and used the cluster stopping rule to aid in selecting partitions [20].

### HRQoL

The Assessment of Quality of Life (AQoL-4D) instrument was used to measure quality of life due to its brevity [21], sensitivity and robustness [22]. Four dimensions (independent living, mental health, relationships and senses) consisting of three items each were included for scoring. Then, five new variables, four dimension scores and one overall instrument score, which ranged from −0.04 to 1, were created. A score of 1.00 indicated the best quality of life equal to perfect health, and 0.00 indicated quality of life equal to death, and negative values (0 to −0.04) indicated quality of life worse than death [23].

### Covariates

Univariate analyses with a 0.25 *p*-value cut-off were performed to screen the covariates before the second round of screening, involving multivariate analyses. A cut-off of a 10% change in the exposure variable’s coefficient estimate in the multivariate model was adopted to identify the “important” variables influencing the association between outcome and exposure. Covariates that remained after these procedures were utilized throughout all subsequent analyses conducted in this study.

The covariates screened in this study included sex, age, registered marital status (married, unmarried), labour force status (employed, unemployed, not in the labour force), area of relative socioeconomic disadvantage (decile 1 = most disadvantaged, decile 10 = least disadvantaged), body mass index (BMI = self-reported weight/self-reported height^2^), smoking status (current, ex-smoker, never) and level of exercise (low: <1600 min; moderate: 1600–3200 min, or >3200 min but <2 h of vigorous exercise; high: >3200 min, including ≥2 h of vigorous exercise), which was also used to assess exercise intensity (exercise intensity scores were multiplied by minutes per fortnight) [11].

### Statistical analyses

Due to the complex survey design used in the NSMHWB2007, a weighting strategy was applied to infer results for the total in-scope population by allocating a ‘weight’ to each sample unit. The weight was an indication of how many population units were represented by the sample unit [11]. As a result, Jack-knife delete-a-group survey adjustment replication methods were used to calculate the standard errors (SEs) [24]. This process accounted for the stratified multistage sampling framework used in the NSMHWB2007 and adjusted for non-response, which may cause some groups to be over- or under-represented [25]. The theory behind the Jack-knife delete-A-group replication method is that, the sampling variability between repeated samples can be estimated by repeatedly taking random but unbiased sub-samples from the achieved sample and then computing the variance of the sub-samples (after taking the smaller sample size into account). Jack-knife estimation replicates are created by deleting one group at a time, and then weighting the other groups from the same stratum to adjust for the removal. Therefore, each replicate provides an unbiased estimate of the population mean, and the variance of those estimates provides an estimate of the full-sample of the variance. In short, application of these methods ensures the sample is representative of the Australian population, which ensures that subsequent findings are generalizable to Australian adults (*n* = 16,015,000) in 2007 [11].

Frequencies and percentages calculated with jack-knife SEs were used for the descriptive analysis. Hierarchical clustering analysis was used to identify common clusters of multiple chronic conditions. Linear regression models were used to examine the associations between the HRQoL scores and the multimorbidity clusters. In each regression model, the dependent variable was the HRQoL score, and the independent variable was one cluster (present or absent), for example, “whether presenting 2+ chronic conditions” in model-1. The p value for the trend of continuous variables in the linear regression models was given. A log-transformed HRQoL score was used due to its negatively skewed distribution, which resulted in 55 missing values. A two-tailed *p*-value of <0.05 was considered statistically significant. To test the validation in clusters of hierarchical clustering, sensitivity analyses were performed that including factor analysis [26], principal component analysis [27] and K-means clustering [28], which have been used in previous studies. All analyses were performed using Stata/SE Version 12.1 (StataCorp, College Station, TX, USA).

## Results

We analysed data from 8841 respondents which could be generalizable to 16,015,000 Australian adults. The mean age of the study participants was 44 years (SE = 0.04). A total of 20.5% (SE = 0.6) of the population was obese (BMI >30), 65.2% (SE = 0.2) were employed, 72.7% (SE = 0.9) reported low levels of exercise, 53% (SE = 0.7) were married and 22.3% (SE = 0.7) were current smokers. More than half of the respondents (56.7%, SE = 0.7) had at least one chronic condition, and 46% had two or more chronic conditions (Table 1).

**Table 1:** Demographic characteristics of the study population, weighted (*N* = 8820)

	*n*	%	Jack-knife standard error
Sex			
Male	4018	49.7	0.01
Female	4802	50.4	0.01
Age			
Mean of age (yr.)	44		0.04
16-25 years.	1551	17.4	0.3
26-35 years.	1386	18.0	0.3
36-45 years.	1586	19.0	0.4
46-55 years.	1247	17.5	0.3
56-65 years.	1293	14.2	0.2
66-75 years.	1069	8.7	0.2
76-85 years.	688	5.2	0.1
BMI (kg/m^2^)			
Thinness (BMI < 18.5)	254	2.7	0.2
Normal (BMI 18.5–24.99)	3701	42.1	0.8
Overweight (BMI 25.00–29.99)	2965	34.7	0.7
Obesity (BMI >30.00)	1724	20.5	0.6
Educational attainment			
Has post-school qualification	4914	54.8	0.5
No post-school qualification	3906	45.2	0.5
Labour force			
Employed	5491	65.2	0.2
Unemployed	215	2.6	0.1
Not in the labour force	3114	32.2	0.2
Level of exercise			
High	597	7.1	0.4
Moderate	1754	20.2	0.7
Low	6463	72.7	0.9
Registered marital status			
Unmarried	3996	47.0	0.7
Married	4824	53.0	0.7
Smoking status			
Current smoker	1875	22.3	0.7
Ex-smoker	2513	26.9	0.7
Never smoked	4432	50.8	0.7
Index of Socio-Economic Disadvantage - Area deciles			
1st decile (lowest)	735	8.1	0.6
2nd decile	789	8.6	0.8
3rd decile	975	10.4	0.8
4th decile	777	8.1	0.8
5th decile	897	9.7	0.8
6th decile	890	10.3	0.9
7th decile	862	10.0	1.0
8th decile	989	11.8	1.0
9th decile	896	11.0	0.8
10th decile (best)	1010	12.0	0.9
Number of health conditions			
0	3556	43.3	0.8
1	2725	30.7	0.8
2	1501	15.8	0.5
3	697	6.8	0.3
4	239	2.5	0.3
5	79	0.6	0.08
6	20	0.2	0.06
7	3	0.02	0.01

*BMI* Body Mass Index. Sample size (n) are showed based on the raw data, proportion (%) are estimated with standard error based on the weighting strategy

Table 2 presents the prevalence of each chronic condition and the percentage of coexistence with other chronic conditions. CVD (21.2%, SE = 0.7), arthritis (19.9%, SE = 0.6) and asthma (19.6%, SE = 0.5) were the three most prevalent conditions. All chronic conditions coexisted with other chronic conditions to various degrees (range from 49 to 91%). Table 3 presents the two common clusters obtained using hierarchical clustering, CVD/arthritis (cluster-1, prevalence = 9.2%, SE = 0.5) and MDD/anxiety (cluster-2, 4.3%, SE = 0.3). In contrast, the prevalence of multimorbidity as defined by the MM2+ and MM3+ count method were 26% (SE = 0.6) and 10.1% (SE = 0.5), respectively.

**Table 2:** Prevalence of single chronic conditions and the percentage with other listed chronic conditions, weighted (*N* = 8,820)

Chronic conditions	*n*	%	Jack-knife standard error	% with other listed conditions
Asthma	1783	19.6	0.5	49.0
Cancer	882	8.3	0.4	70.6
Stroke	234	2.0	0.1	91.6
CVD	2059	21.2	0.7	69.9
Arthritis	1989	19.9	0.6	72.7
Diabetes	700	7.5	0.4	77.5
MDD	658	7.2	0.4	82.3
Anxiety Disorder	1005	11.4	0.4	71.0

*CVD* Heart or circulatory condition, *MDD* Major depression disorder, Sample size (n) are showed based on the raw data, proportion (%) are estimated with standard error based on the weighting strategy

**Table 3:** Prevalence of common clusters using count method and hierarchical cluster, weighted (*N* = 8,820)

	Components	*n*	Prevalence (%)	Jack-knife standard error	Methods
MM2+	any 2 (+) of 8 chronic conditions^a^	2539	26.0	0.6	Count method
MM3+	any 3 (+) of 8 chronic conditions^b^	1042	10.1	0.5	Count method
Cluster 1	CVD/Arthritis	934	9.1	0.5	Hierarchical cluster
Cluster 2	MDD/Anxiety Disorder	399	4.3	0.3	Hierarchical cluster

*CVD* Heart or circulatory condition, *MDD* Major depression disorder, *MM* multimorbidity. Sample size (n) are showed based on the raw data, proportion (%) are estimated with standard error based on the weighting strategy

^a^MM2+ which means having any 2 or more chronic conditions out of asthma, cancer, stroke, CVD, gout rheumatism or arthritis and diabetes or high sugar levels, MDD and anxiety disorder

^b^MM3+ which means having any 3 or more chronic conditions out of asthma, cancer, stroke, CVD, gout rheumatism or arthritis and diabetes or high sugar levels, MDD and anxiety disorder

The mean ages of the population with MM2+, MM3+, cluster-1 and cluster-2 were 54.6 (SE = 0.3), 57.5 (SE = 0.6), 63.8 (SE = 0.7) and 41.7 (SE = 0.7) years, respectively. As expected, prevalence of MM3+ was lower than MM2+ (Table 3), but mean HRQoL was poorer. Interestingly both count methods identified groups with similar socio-demographic characteristics such as female, older, higher BMI, lower education level, less exercise, lower socio-economic status, and not in the labour force. Individuals with MDD/anxiety (cluster-2), which resulting from hierarchical clustering to identify multimorbidity, had the lowest HRQoL scores with the different socio-demographic characteristics comparing to the other hierarchical cluster and count method to identify multimorbidity, such as younger, unemployed, unmarried (Tables 4–5).

**Table 4:** Mean of AQoL-4D utility scores by sample characteristics using count method to identify multimorbidity, weighted (*N* = 8,820)

	MM2 + ^a^			MM3 + ^b^		
	%	Mean	Jack-knife standard error	%	Mean	Jack-knife standard error
Sex						
Male	23.8	0.72	0.01	8.7	0.65	0.02
Female	27.9	0.69	0.01	11.4	0.61	0.01
Age						
Mean of age (yr.)		54.6	0.3		57.5	0.6
16-25 years.	8.9	0.68	0.03	2.4	0.56	0.09
26-35 years.	14.1	0.67	0.02	3.3	0.54	0.05
36-45 years.	18.7	0.67	0.03	6.9	0.60	0.07
46-55 years.	26.6	0.68	0.02	9.5	0.57	0.03
56-65 years.	42.2	0.73	0.01	18.7	0.66	0.02
66-75 years.	52.8	0.75	0.01	23.1	0.70	0.02
76-85 years.	58.5	0.70	0.02	27.7	0.63	0.02
BMI (kg/m^2^)						
Thinness (BMI < 18.5)	19.2	0.63	0.06	5.3	0.34	0.10
Normal (BMI 18.5-24.99)	20.3	0.71	0.01	6.2	0.66	0.02
Overweight (BMI 25.00-29.99)	25.7	0.71	0.01	10.8	0.64	0.03
Obesity (BMI >30.00)	38.4	0.70	0.01	17.4	0.60	0.02
Educational attainment						
Has post-school qualification	23.8	0.72	0.01	8.5	0.66	0.02
No post-school qualification	28.4	0.69	0.01	12.1	0.60	0.02
Labour force						
Employed	18.2	0.74	0.01	5.6	0.64	0.02
Unemployed	20.5	0.63	0.04	8.7	0.60	0.07
Not in the labour force	42.0	0.68	0.01	19.4	0.62	0.02
Level of exercise						
High	17.2	0.75	0.02	7.1	0.67	0.04
Moderate	23.1	0.77	0.02	8.0	0.69	0.04
Low	27.5	0.69	0.01	10.9	0.61	0.01
Registered marital status						
Unmarried	22.9	0.64	0.01	9.1	0.57	0.02
Married	28.4	0.75	0.01	10.9	0.67	0.02
Smoking status						
Current smoker	26.5	0.62	0.02	10.7	0.53	0.03
Ex-smoker	32.8	0.72	0.01	14.2	0.66	0.02
Never smoked	21.8	0.74	0.01	7.6	0.66	0.02
Index of Socio-Economic Disadvantage - Area deciles						
1st decile (lowest)	29.9	0.62	0.03	15.8	0.54	0.05
2nd decile	27.1	0.66	0.03	10.6	0.62	0.03
3rd decile	29.1	0.65	0.02	13.6	0.59	0.03
4th decile	28.9	0.66	0.03	10.8	0.59	0.03
5th decile	29.8	0.70	0.02	13.0	0.64	0.03
6th decile	25.5	0.74	0.02	9.5	0.70	0.07
7th decile	26.1	0.72	0.03	9.4	0.64	0.04
8th decile	24.1	0.76	0.02	6.8	0.66	0.03
9th decile	21.0	0.76	0.02	6.6	0.67	0.03
10th decile (best)	20.6	0.77	0.02	7.2	0.67	0.04

*CVD* Heart or circulatory condition, *MDD* Major depression disorder, *BMI* Body Mass Index, *MM* multimorbidity. Means are estimated with standard error based on the weighting strategy

^a^MM2 + which means having any 2 or more chronic conditions out of asthma, cancer, stroke, CVD, gout rheumatism or arthritis and diabetes or high sugar levels, MDD and anxiety disorder

^b^MM3 + which means having any 3 or more chronic conditions out of asthma, cancer, stroke, CVD, gout rheumatism or arthritis and diabetes or high sugar levels, MDD and anxiety disorder

**Table 5:** Mean of AQoL-4D utility scores by sample characteristics using hierarchical cluster to identify multimorbidity, weighted (*N* = 8820)

	Cluster-1^a^			Cluster-2^b^		
	%	Mean	Jack-knife standard error	%	Mean	Jack-knife standard error
Sex						
Male	8.6	0.75	0.02	3.7	0.54	0.04
Female	9.6	0.68	0.01	4.8	0.52	0.02
Age						
Mean of age (yr.)		63.8	0.7		41.7	0.7
16-25 years.	0.4	0.41	0.40	3.4	0.57	0.03
26-35 years.	0.6	0.72	0.10	4.6	0.57	0.04
36-45 years.	3.6	0.75	0.12	6.9	0.54	0.05
46-55 years.	6.6	0.68	0.03	4.7	0.49	0.05
56-65 years.	20.3	0.72	0.02	3.4	0.44	0.06
66-75 years.	28.3	0.75	0.01	2.0	0.53	0.05
76-85 years.	34.7	0.66	0.02	0.6	0.24	0.13
BMI (kg/m^2^)						
Thinness (BMI < 18.5)	5.9	0.58	0.17	5.5	0.40	0.10
Normal (BMI 18.5-24.99)	4.8	0.73	0.02	4.5	0.60	0.03
Overweight (BMI 25.00-29.99)	9.5	0.73	0.02	3.4	0.47	0.04
Obesity (BMI >30.00)	17.8	0.68	0.02	4.8	0.48	0.03
Educational attainment						
Has post-school qualification	7.7	0.75	0.01	4.2	0.54	0.03
No post-school qualification	10.8	0.67	0.02	4.3	0.51	0.03
Labour force						
Employed	4.0	0.76	0.02	3.9	0.60	0.02
Unemployed	2.0	0.67	0.18	8.2	0.53	0.07
Not in the labour force	20.2	0.69	0.02	4.7	0.40	0.03
Level of exercise						
High	4.1	0.74	0.04	3.5	0.61	0.04
Moderate	7.8	0.82	0.03	4.4	0.50	0.05
Low	10.0	0.69	0.01	4.3	0.53	0.02
Registered marital status						
Unmarried	6.5	0.64	0.02	5.9	0.49	0.02
Married	11.4	0.75	0.02	2.8	0.59	0.05
Smoking status						
Current smoker	5.4	0.61	0.05	8.4	0.49	0.03
Ex-smoker	14.7	0.72	0.02	3.6	0.54	0.05
Never smoked	7.8	0.74	0.02	2.8	0.56	0.03
Index of Socio-Economic Disadvantage - Area deciles						
1st decile (lowest)	11.5	0.59	0.06	5.9	0.44	0.06
2nd decile	9.7	0.69	0.03	3.1	0.46	0.05
3rd decile	11.0	0.66	0.03	5.6	0.42	0.06
4th decile	9.1	0.65	0.04	6.4	0.53	0.09
5th decile	11.8	0.69	0.03	5.6	0.47	0.06
6th decile	9.2	0.81	0.05	3.5	0.59	0.04
7th decile	10.7	0.76	0.03	4.1	0.56	0.05
8th decile	7.2	0.78	0.03	3.8	0.65	0.08
9th decile	5.3	0.77	0.04	3.0	0.59	0.07
10th decile (best)	7.2	0.74	0.05	2.7	0.63	0.05

*CVD* Heart or circulatory condition, *MDD* Major depression disorder, *BMI* Body Mass Index, *MM* Multimorbidity. Means are estimated with standard error based on the weighting strategy

^a^Cluster-1 = CVD + Arthritis

^b^Cluster-2 = MDD + Anxiety Disorder

Individuals with MDD/anxiety (cluster-2) had HRQoL scores that were 0.38 points (SE = 0.02; *p* < 0.01) lower than those without cluster-2. Individuals with any two or more chronic conditions (MM2+) had HRQoL scores that were 0.21 points (SE = 0.01; *p* < 0.01) lower than those with no more than one chronic condition. Individuals with any three or more chronic conditions (MM3+) had HRQoL scores that were 0.26 points (SE = 0.02; *p* < 0.01) lower than those with no more than two chronic conditions. Individuals with CVD/arthritis (cluster-1) had HRQoL scores that were 0.14 points lower than those without CVD/arthritis. After adjusting for sex, age, BMI, labour force status, level of exercise (not in the model of cluster-2), registered marital status, smoking status and socio-economic disadvantage index, multivariate analyses revealed the associations between the HRQoL scores and each cluster remained significant; the MM2+ cluster (coef: −0.18, SE = 0.01; *p* < 0.01) and the MM3+ cluster (coef: −0.23, SE = 0.02; *p* < 0.01) were higher than the CVD/arthritis cluster (coef: −0.10, SE = 0.01; *p* < 0.001) but lower than the MDD/anxiety cluster (coef: −0.36, SE = 0.01; *p* < 0.001) (Tables 6–7).

**Table 6:** Mean of AQoL-4D utility scores and linear associations by sample characteristics using count method to identify multimorbidity, weighted

			Model 1 – Present. MM2 + ^a^		Model 2 – Present. MM3 + ^b^	
	Univariate analysis		Multivariate analysis		Multivariate analysis	
	Unadjusted β^c^	Jack-knife standard error	Adjusted β^d^	Jack-knife standard error	Adjusted β^d^	Jack-knife standard error
**Sex**						
Male	Ref.		Ref.		Ref.	
Female	−0.02	0.01	0.001	0.01	0.002	0.01
Age						
16-25 years.	Ref.		Ref.		Ref.	
26-35 years.	**−0.04**	**0.01**	**−0.05**	**0.01**	**−0.06**	**0.01**
36-45 years.	**−0.05**	**0.01**	**−0.07**	**0.01**	**−0.08**	**0.01**
46-55 years.	**−0.08**	**0.02**	**−0.09**	**0.01**	**−0.10**	**0.01**
56-65 years.	**−0.09**	**0.01**	**−0.04**	0.02	**−0.06**	**0.02**
66-75 years.	**−0.09**	**0.01**	0.02	0.02	−0.01	0.02
76-85 years.	**−0.19**	**0.02**	−0.05	0.03	**−0.08**	**0.03**
p for trend	**<0.05**		=0.20		**<0.05**	
BMI (kg/m^2^)						
Thinness (BMI < 18.5)	−0.08	0.05	−0.01	0.01	−0.05	0.03
Normal (BMI 18.5-24.99)	Ref.		Ref.			
Overweight (BMI 25.00-29.99)	0.03	0.01	−0.02	0.01	−0.01	0.01
Obesity (BMI >30.00)	**−0.01**	**0.02**	**−0.02**	**0.01**	**−0.02**	**0.01**
p for trend	**<0.05**		=0.30		=0.30	
Labour force status						
Employed	Ref.		Ref.		Ref.	
Unemployed	−0.05	0.03	−0.02	0.03	−0.02	0.03
Not in the labour force	**−0.13**	**0.01**	**−0.11**	**0.02**	**−0.10**	**0.02**
Level of exercise						
High	Ref.		Ref.		Ref.	
Moderate	−0.003	0.02	0.01	0.02	0.004	0.02
Low	**−0.06**	**0.02**	−0.03	0.02	−0.03	0.02
Registered marital status						
Unmarried	Ref.		Ref.		Ref.	
Married	**0.05**	**0.01**	**0.08**	**0.01**	**0.08**	**0.01**
Smoking status						
Current smoker	Ref.		Ref.		Ref.	
Ex-smoker	**0.04**	**0.01**	**0.04**	**0.02**	**0.04**	**0.01**
Never smoked	**0.09**	**0.01**	**0.06**	**0.01**	**0.06**	**0.01**
Index of Socio-Economic Disadvantage - Area deciles						
1st decile (lowest)	Ref.		Ref.		Ref.	
2nd decile	0.01	0.02	−0.002	0.02	−0.01	0.02
3rd decile	−0.01	0.02	−0.03	0.02	−0.03	0.02
4th decile	0.01	0.03	−0.01	0.02	−0.01	0.02
5th decile	0.03	0.02	0.002	0.02	−0.003	0.02
6th decile	0.03	0.02	−0.01	0.02	−0.02	0.02
7th decile	**0.05**	0.02	0.02	0.02	0.01	0.02
8th decile	**0.07**	0.02	0.02	0.02	0.01	0.02
9th decile	**0.07**	0.03	0.01	0.02	0.01	0.02
10th decile (best)	**0.09**	0.02	0.02	0.02	0.01	0.02
p for trend	**<0.05**		**<0.05**		**<0.05**	
Present. MM2 + ^a^	**−0.21**	**0.01**	**−0.18**	**0.01**		
Present. MM3 + ^a^	**−0.26**	**0.02**			**−0.23**	**0.02**
Observations			8605		8605	
Weighted R^2^			**0.1219**		**0.1159**	

*BMI* Body Mass Index

Means are estimated with standard error based on weighting strategy

^a^ MM2 + which means having any 2 or more chronic conditions out of asthma, cancer, stroke, CVD, gout rheumatism or arthritis and diabetes or high sugar levels, MDD and anxiety disorder

^b^ MM3 + which means having any 3 or more chronic conditions out of asthma, cancer, stroke, CVD, gout rheumatism or arthritis and diabetes or high sugar levels, MDD and anxiety disorder

^c^ β from univariate linear regression for the associations with HRQoL

^d^ β from multivariate linear regression model with all other variables in the table adjusted for the associations with HRQoL

Significant coefficients are typed in bold font (*p*< 0.05)

**Table 7:** Mean of AQoL-4D utility scores and linear associations by sample characteristics using hierarchical cluster to identify multimorbidity, weighted

			Model 3 – Cluster-1^a^		Model 4 – Cluster-2^a^	
	Univariate analysis		Multivariate analysis		Multivariate analysis	
	Unadjusted β^b^	Jack-knife standard error	Adjusted β^c^	Jack-knife standard error	Adjusted β^c^	Jack-knife standard error
**Sex**						
Male	Ref.		Ref.		Ref.	
Female	−0.02	0.01	0.01	0.01	0.01	0.01
Age						
16-25 years.	Ref.		Ref.		Ref.	
26-35 years.	**−0.04**	**0.01**	**−0.06**	**0.01**	**−0.05**	**0.01**
36-45 years.	**−0.05**	**0.01**	**−0.09**	**0.01**	**−0.08**	**0.01**
46-55 years.	**−0.08**	**0.02**	**−0.13**	**0.01**	**−0.12**	**0.01**
56-65 years.	**−0.09**	**0.01**	**−0.08**	**0.01**	**−0.09**	**0.01**
66-75 years.	**−0.09**	**0.01**	−0.03	0.02	**−0.06**	**0.02**
76-85 years.	**−0.19**	**0.02**	**−0.08**	**0.02**	**−0.12**	**0.02**
p for trend	**<0.05**		**<0.05**		**<0.05**	
BMI (kg/m^2^)						
Thinness (BMI < 18.5)	−0.08	0.05	−0.05	0.02	−0.05	0.02
Normal (BMI 18.5-24.99)	Ref.		Ref.		Ref.	
Overweight (BMI 25.00-29.99)	0.03	0.01	−0.01	0.01	−0.02	0.01
Obesity (BMI >30.00)	**−0.01**	**0.02**	**−0.05**	**0.01**	**−0.05**	**0.01**
p for trend	**<0.05**		**<0.05**		**<0.05**	
Labour force status						
Employed	Ref.		Ref.		Ref.	
Unemployed	−0.05	0.03	−0.06	0.02	−0.04	0.02
Not in the labour force	**−0.13**	**0.01**	**−0.12**	**0.01**	**−0.11**	**0.01**
Level of exercise^d^						
High	Ref.		Ref.			
Moderate	−0.003	0.02	0.01	0.02		
Low	**−0.06**	**0.02**	−0.03	0.02		
Registered marital status						
Unmarried	Ref.		Ref.		Ref.	
Married	**0.05**	**0.01**	**0.09**	**0.01**	**0.07**	**0.01**
Smoking status						
Current smoker	Ref.		Ref.		Ref.	
Ex-smoker	**0.04**	**0.01**	**0.05**	**0.01**	**0.04**	**0.01**
Never smoked	**0.09**	**0.01**	**0.07**	**0.01**	**0.06**	**0.01**
Index of Socio-Economic Disadvantage - Area deciles						
1st decile (lowest)	Ref.		Ref.		Ref.	
2nd decile	0.01	0.02	0.002	0.02	0.01	0.02
3rd decile	−0.01	0.02	0.001	0.02	0.01	0.02
4th decile	0.01	0.03	0.01	0.02	0.02	0.02
5th decile	0.03	0.02	0.02	0.02	0.02	0.02
6th decile	0.03	0.02	0.01	0.02	0.02	0.02
7th decile	**0.05**	**0.02**	0.02	0.02	0.03	0.02
8th decile	**0.07**	**0.02**	0.04	0.02	0.04	0.02
9th decile	**0.07**	**0.03**	0.03	0.02	0.04	0.02
10th decile (best)	**0.09**	**0.02**	0.04	0.02	0.05	0.02
p for trend	**<0.05**		**<0.05**		**<0.05**	
Present. Cluster1^a^	**−0.14**	**0.02**	**−0.10**	**0.01**		
Present. Cluster2^a^	**−0.38**	**0.02**			**−0.36**	**0.01**
Observations			8605		8605	
Weighted R^2^			**0.0773**		**0.1427**	

*CVD* Heart or circulatory condition, *MDD* Major depression disorder, *BMI* Body Mass Index, *MM* multimorbidity

^a^Cluster-1 = CVD + Arthritis; Cluster-2 = MDD + Anxiety Disorder

^b^β from univariate linear regression for the associations with HRQoL

^c^β from multivariate linear regression models with all other variables in the table adjusted for the associations with HRQoL

^d^“level of exercise” was not included in model-4 because it was excluded in confounder selection

Significant coefficients are typed in bold font (*p*< 0.05)

## Discussion

Consistent with the findings of the 2004 systematic review by Fortin et al. [8], our analysis of a large, nationally representative dataset showed that multimorbidity is common and significantly associated with lower HRQoL. Although the different definitions of multimorbidity did not change this association, the sociodemographic profiles and HRQoL scores varied depending on the definition of multimorbidity. In the present study, the HRQoL scores were lowest in the participants characterized by cluster-2 (MDD/anxiety disorders), followed by MM2+, which defined multimorbidity as 2+ condition entities, and cluster-1 (CVD/arthritis).

Although this study failed to identify a specific cluster of comorbid mental and physical disorders, previous research has demonstrated that the co-occurrence of mental and physical disorders is strongly associated with poorer HRQoL [29]. Therefore, the findings of this study suggest that the count method does not take the type of chronic conditions into account. Therefore, this method can detect the overall influence of multimorbidity on the HRQoL, but it does not capture the specific disease that contributes to the associated HRQoL.

Individual disease-based treatments can help relieve associated discomfort, slow the course of disease and increase the HRQoL for people with a single chronic condition. However, for individuals with multimorbidity, traditional, individual, disease-focused treatment does not perform well due to interactions between the diseases and treatments. Moreover, reducing the number of conditions does not provide health professionals with an effective therapeutic plan. Furthermore, when calculating the burden of multimorbidity, the condition needs to be treated in its entirety if it is to inform accurate health care planning.

Different cut-off values of the number-based count definition of multimorbidity have been used in the previous HRQoL studies [30]. Some of them used both two or more (2+) and three or more (3+) chronic conditions at the same time [15, 31–33] as the cut off value. Harrison C, Britt H, Miller G and Henderson J [15] reported that the 2+ definition was more appropriate in a broader age-scope population, whereas 3+ was more specific for an elderly study population [15]. However, no cut-off can be used without caution, particularly because the number of conditions in the current studies ranged from 4 to 102 [33, 34]. Furthermore, the 2+ cut-off is recommended when a limited number of chronic conditions are included in the definition of multimorbidity, whereas the 3+ cut-off requires the inclusion of more chronic conditions [15]. Despite these issues, the 2+ cut-off was deemed most appropriate for our study based on the data used, i.e., eight chronic conditions and a population-based sample.

In addition to hierarchical clustering, other approaches to the common clusters of multimorbidity exist including factor analysis, principal component analysis and K-means clustering [16]. This study used hierarchical clustering with Jaccard’s coefficient due to the shared risk factors among the chronic conditions and the binary nature of chronic diseases [19]. However, the other three approaches were tested in a sensitivity analysis using the same sample (results not shown). The same clusters were produced by the factor analysis and principal component analysis: CVD/arthritis (cluster-1) and cancer/stroke/CVD/arthritis/diabetes. K-means analysis produced clusters including cancer/stroke/CVD/arthritis/diabetes and cancer/stroke/CVD/diabetes/MDD/anxiety. Cancer/stroke/CVD/arthritis/diabetes and cancer/stroke/CVD/diabetes/MDD/anxiety were not examined further due to the extremely low prevalence, with only five and two cases, respectively. These differences may be due to the different mechanisms of the methods used to detect the clusters, i.e., the cluster analysis processes used distance measures, whereas the factor analysis and principal component analysis processes used correlations. In addition, the individuals within these distance-based clusters have more common characteristics. Furthermore, because the results of the hierarchical cluster approach may be sensitive to the distance scales and linking methods, we performed sensitivity analyses using an additional four distance scales: Ward’s linkage, waverage linkage, single linkage and complete linkage. All of these scales produced the same results as average linkage, except for single linkage. To test the sensitivity of different cut-off of count-based method, MM3+ as the complex multimorbidity in literature was used as well. [15] The results shown that even prevalence of multimorbidity as well as the mean HRQoL scores in the people considered as multimorbid varied by the different cut-off of multimorbidity used, multivariate analyses revealed similar patterns in the variations of estimates of HRQoL scores within each of the subgroups of individuals considered. In relation to the cluster definitions of multimorbidity, the method does not pre-specify number of conditions but is statistically derived, thus these analyses remain unchanged.

This study has several notable limitations. First, the findings of multimorbidity studies must be in considered with reference to the list of conditions included in the definition of multimorbidity, as the prevalence of multimorbidity depends on the definition used. [34] However, the health conditions used in this study were chosen because they contribute significantly to the burden of disease in the Australian community. Moreover, the present study excluded acute conditions, which some previous studies have included [6]. Although including more conditions in the definition of multimorbidity may potentially provide a more comprehensive understanding of an individual’s health status, acute conditions were not considerate in this study as they may only influence health status temporarily [15] and not be relevant to long-term health care planning.

Second, the data used in this study were derived from a survey focused on mental health well-being. As a result, the assessments of physical chronic conditions were relatively brief, self-reported and not verified using medical records [11]. Physical conditions were assessed by self-report in the past 12-months, which may be underestimated or overestimated due to recall bias. However, the validity of self-reported chronic conditions has been indicated in different contexts [35–39]. Moreover, self-reported data offers cost-effectiveness and convenience for gathering information in the population-based surveys. [40] Finally, despite being encouraged [32], the severity of chronic conditions was not included in this study because it was not measured in the NSMHWB2007 and the additional burden on the respondents (time consuming) may reduce the response rate. Although it is unlikely to change the present status of the condition when defining multimorbidity, the severity may have influenced the HRQoL scores.

To our knowledge, this is the first study to compare number-based and cluster-based definitions of multimorbidity using nationally representative data. This large population-based database, using the delete-1 group jack-knife technique to generate the replicate weights, increases the generalizability of the study’s findings and could inform the investigation of multimorbidity-related HRQoL in Australia and similar economies worldwide.

## Conclusions

Our findings confirm the existence of an inverse relationship between multimorbidity and HRQoL in the Australian population and indicate that the sociodemographic profile and HRQoL vary depending on the method used to define multimorbidity. We conclude that from this head-to-head comparison, the hierarchical clustering approach has been validated when the outcome of interest is HRQoL. Moreover, a simple count fails to identify if there are specific conditions of interest that are driving lower HRQoL. From this perspective, the cluster-based methods, resulting in clusters with the same shared health conditions, may be more useful and informative. Finally, we recommend that researchers exercise caution when selecting a definition of multimorbidity because it may significantly influence the study outcomes.

## Abbreviations

ABS: Australian Bureau of Statistics
AQoL-4D: Assessment of Quality of Life-4D
BMI: body mass index
CVD: heart or circulatory condition
GAD: generalized anxiety disorder
HRQoL: Health-related Quality of Life
MDD: major depressive disorder
MM: multimorbidity
NSMHWB: Australian National Survey of Mental Health and Wellbeing
SE: standard error
WMH-CIDI 3.0: The World Mental Health Survey Initiative version of the World Health Organization's Composite International Diagnostic Interview, version 3.0
